# Urban specialization reduces habitat connectivity by a highly mobile wading bird

**DOI:** 10.1186/s40462-020-00233-7

**Published:** 2020-12-07

**Authors:** Claire S. Teitelbaum, Jeffrey Hepinstall-Cymerman, Anjelika Kidd-Weaver, Sonia M. Hernandez, Sonia Altizer, Richard J. Hall

**Affiliations:** 1 Odum School of Ecology, University of Georgia, Athens, GA USA; 2 Warnell School of Forestry and Natural Resources, University of Georgia, Athens, GA USA; 3 Present address: College of Agriculture, Forestry and Life Sciences, Clemson University, Clemson, SC USA; 4 Southeastern Cooperative Wildlife Disease Study, College of Veterinary Medicine, University of Georgia, Athens, GA USA; 5 Department of Infectious Diseases, College of Veterinary Medicine, University of Georgia, Athens, GA USA

**Keywords:** American white ibis (*Eudocimus albus*), Connectivity, Habitat specialization, Network, Nomadism, Urbanization, Wildlife

## Abstract

**Background:**

Mobile animals transport nutrients and propagules across habitats, and are crucial for the functioning of food webs and for ecosystem services. Human activities such as urbanization can alter animal movement behavior, including site fidelity and resource use. Because many urban areas are adjacent to natural sites, mobile animals might connect natural and urban habitats. More generally, understanding animal movement patterns in urban areas can help predict how urban expansion will affect the roles of highly mobile animals in ecological processes.

**Methods:**

Here, we examined movements by a seasonally nomadic wading bird, the American white ibis (*Eudocimus albus*), in South Florida, USA. White ibis are colonial wading birds that forage on aquatic prey; in recent years, some ibis have shifted their behavior to forage in urban parks, where they are fed by people. We used a spatial network approach to investigate how individual movement patterns influence connectivity between urban and non-urban sites. We built a network of habitat connectivity using GPS tracking data from ibis during their non-breeding season and compared this network to simulated networks that assumed individuals moved indiscriminately with respect to habitat type.

**Results:**

We found that the observed network was less connected than the simulated networks, that urban-urban and natural-natural connections were strong, and that individuals using urban sites had the least-variable habitat use. Importantly, the few ibis that used both urban and natural habitats contributed the most to connectivity.

**Conclusions:**

Habitat specialization in urban-acclimated wildlife could reduce the exchange of propagules and nutrients between urban and natural areas, which has consequences both for beneficial effects of connectivity such as gene flow and for detrimental effects such as the spread of contaminants or pathogens.

## Background

Habitat connectivity, defined as the movement of organisms and materials among patches on landscapes [1], is important for ecological processes and their outcomes, including population viability, genetic structure, infection dynamics, and ecosystem services [2–7]. By definition, animal movements are a key component of habitat connectivity [8, 9]. Animal movements across ecosystem boundaries can facilitate the transfer of nutrients, seeds, and beneficial microbes between environments that differ in their productivity or community composition [8, 10]. For example, seabirds that move from pelagic to coastal systems in the Pacific Ocean transport nutrients and increase soil nitrogen levels on islands, with further downstream effects that increase nutrient loads in runoff, zooplankton biomass, and manta ray abundance [11]. This functional connectivity of heterogeneous landscapes depends on animals’ movement abilities, distances between patches, patch sizes, resistance or barriers to movement, and other landscape properties [12].

Human activities can alter both the physical properties of landscapes and animal behavior, producing changes in functional connectivity [12]. In the Pacific, seabirds tend to avoid coastlines dominated by human-associated coconut palms, making these areas less connected to pelagic systems and resulting in lower abundances of zooplankton and manta rays [11]. Urbanization is another human activity that alters animal movement by fragmenting landscapes, and also by providing novel resources for species that can adapt or acclimate to urban environments [13]. Although some animals can move easily in urban landscapes, urbanization generally reduces connectivity by increasing the distance between patches and introducing barriers to movement [12, 14]. Species that are habitat generalists and have high movement capacity might be less limited by barriers or fragmentation than habitat specialists [15, 16], but even species that are highly mobile in natural environments often move less in urban areas (e.g., have smaller home ranges, show higher site fidelity, are less likely to migrate seasonally) [17–19]. This reduced movement stems in part from the presence of reliable resources in urban landscapes [20]; for instance, migratory white storks that feed on landfills have recently established resident populations [21] and brown bears revisit known feeding sites in winter when resources are otherwise scarce [22]. These less-frequent or shorter-distance movements can limit connectivity to the extent of creating apparent landscape fragmentation, even when it is not present structurally (i.e., habitat-independent fragmentation: [23]). Highly mobile species can be particularly important for connectivity because they transport propagules over long distances [24], so understanding their movement responses to urbanization is important for understanding both population and ecosystem processes.

Functional connectivity, defined as the connectivity of a landscape from the perspective of a focal organism [25], is often modeled by combining information on patch locations, non-habitat matrix, and average species movements [12, 25, 26]. Many models assume that interpatch distances are the primary determinant of connectivity, using random walks [27] or dispersal kernels [28]. In many cases, movement is assumed to be optimal and equivalent across all individuals [29] (but see [30]). However, individuals often differ in their movement patterns [31, 32], which can complicate estimates of functional connectivity. For example, individuals can specialize in their use of habitat types (e.g., “urban” and “nonurban” individuals) [33, 34], especially in circumstances where urban habitats favor specific behavioral types [13]. This specialization could reduce the frequency of movements across ecosystem boundaries. Conversely, individuals might gain complementary resources from urban and natural areas, which would increase movement rates between habitat types [1]. Incorporating individual variation into habitat selection models substantially improved the accuracy of connectivity estimates for elephants [35]; overall, if habitat selection varies between individuals or if movements are based on habitat type, connectivity could be lower (or higher) than that predicted based on distances alone.

Here, we analyze movements of American white ibis (*Eudocimus albus*) in the southeastern United States to investigate connectivity across a patchy landscape of urban and natural sites. The term “natural” has many definitions [36]; in this paper, we use “natural” to refer to non-urban habitats that maintain the historical structure and function of local ecosystems, even if these habitats are managed or constructed by people (e.g., managed wetlands). White ibis are colonial wading birds that inhabit wetlands, where they feed on fish and aquatic invertebrates [37]. Ibis in these natural areas are nomadic, moving to new foraging and roosting sites in the region when conditions change [38]. Their high mobility means that they could be important for connecting distant patches (ibis can move 30–60 km daily between roosting and foraging sites, [39, 40]). At the same time, urbanization in South Florida is associated with shifts in ibis foraging and movement behavior. Many ibis now forage in city parks, where they feed on human-provided resources (including bread) and show higher site fidelity, returning to the same parks over weeks or months [41, 42]. Past work in this system showed that ibis can be infected by generalist enteric pathogens such as *Salmonella,* for which infection prevalence is highest in urban flocks [43, 44]. Ibis might contribute to *Salmonella* transmission, or their exposure could reflect transmission from other reservoir species or environmental sources [45].

Here, we use a spatial network approach to study ibis movements during the nonbreeding season. Our goals are to better understand animal movement responses to urbanization, and to predict the consequences of these movements for the dispersal of ecosystem services, pathogens and contaminants across urban and natural sites. Specifically, we: (i) build an empirically-derived network representing ibis movements among frequently visited patches over a 3-year period; (ii) compare this observed network to networks based on simulated movements to determine whether ibis movements increase or decrease habitat connectivity; and (iii) examine how individual birds differ in their habitat use and connectivity roles.

We predict that site fidelity and habitat specialization will reduce network connectivity, as measured by edge density, assortativity, and modularity (Table 1). If individuals show minimal site fidelity or habitat specialization, then these three properties will be the same in the observed and simulated networks. In contrast, if individuals show high site fidelity, then edge density will be lower and modularity will be higher in the observed network than in the simulated networks. Further, if individuals specialize in their use of habitat types, then assortativity of land cover will be higher in the observed network than in the simulated networks and modules (defined as groups of nodes that are closely connected: Table 1) will differ in their land cover.

**Table 1:** Network metrics used in this study. In the "Range of values" column, square brackets indicate that a range includes the endpoint and parentheses indicate that a range excludes the endpoint. In each example diagram, nodes of different colors represent different habitat types or land cover classes

Name	Definition	Ecological interpretation	Range of values	Example
***Network-level metrics***				
Edge density	The proportion of potential connections in the network that are realized	Landscape connectedness	(0,1]	
Assortativity	The tendency of nodes with similar properties to be connected to one another	Connectivity among habitats of the same vs. of different types	[-1,1]	
Modularity	The ability of a network to be divided into communities, where there are few edges between communities	Aggregation of groups of patches, “functional spatial structure” [46]	[0,1]	
***Node-level metrics***				
Degree centrality	The number of links of a focal node. In a directed network, can be *in-degree* (the number of incoming links) or *out-degree* (the number of outgoing links)	The potential number of other patches that a contaminant, nutrient, etc. could directly spread to (*out-degree*) or come from (*in-degree*)	(0,N] (N=# of nodes in net-work)	
Betweenness centrality	The fraction of shortest paths between nodes that pass through the focal node	Role of a patch as a “stepping stone” that connects otherwise-separated groups of patches	[0,1]	
Node size	Sum of all edge weights entering and leaving a node	Number of visits to a patch	[1,Infinity)	

We further predict that ibis that more spend time in urban habitats will be site-faithful and show greater habitat specialization, based on observations of ibis and other species in urban areas [5, 42, 47]. Individuals might differ in their roles for connecting the network [48, 49]; in particular, we predict that the least specialized individuals (i.e., those that frequently use both urban and natural sites) will contribute disproportionately to network connectivity.

## Methods

### Field methods

**Fig. 1:**
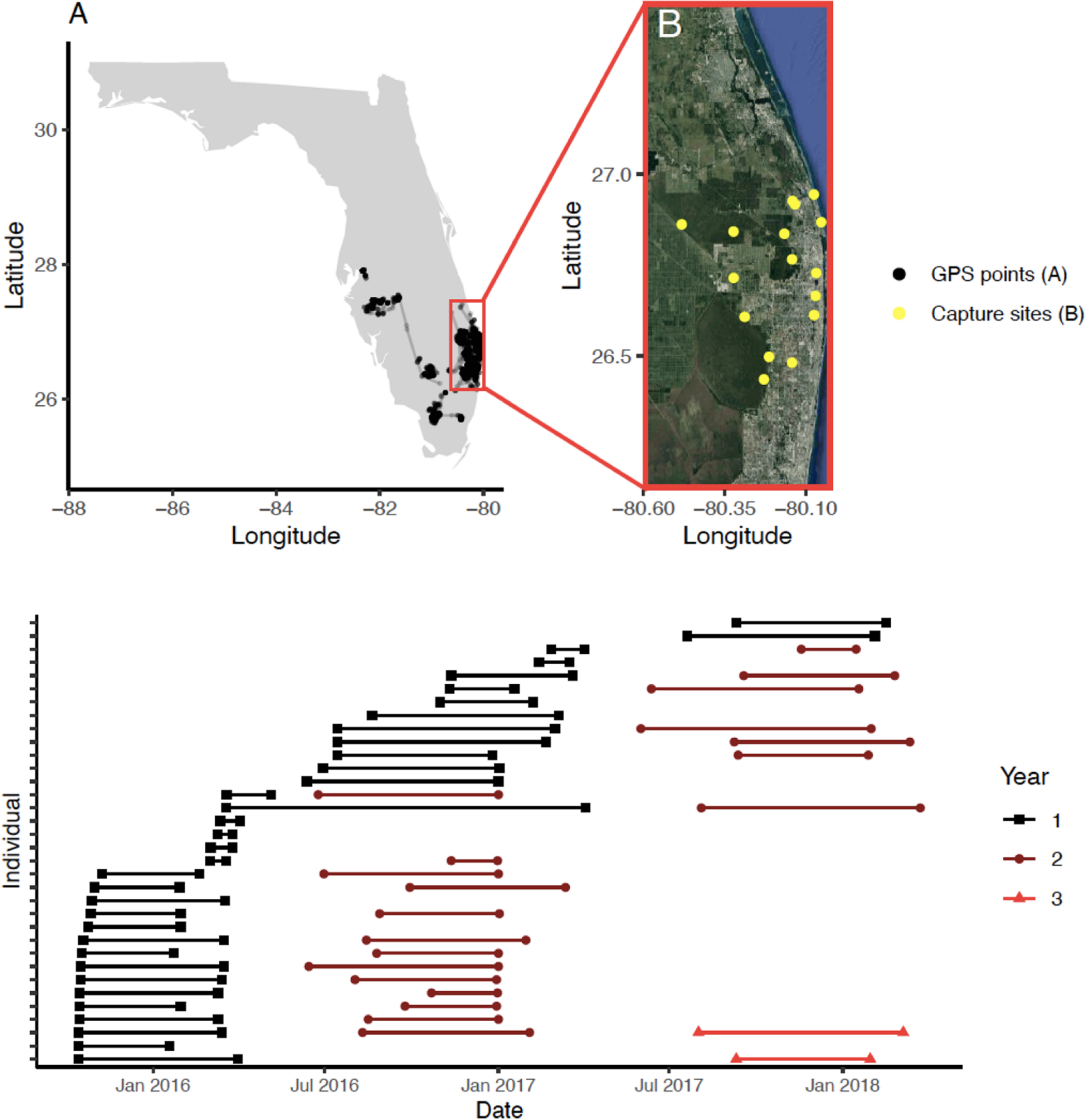
Study area and tracking data used in analyses. **a** All GPS tracking data (*n* = 46,111 points) for the nonbreeding season on a map of Florida, USA. The red outline shows the study area used in analyses. Tracks that fell entirely outside the study area (*n* = 5) were excluded because they were not connected to the core study area during an entire nonbreeding season. **b** Satellite imagery and capture site locations within the study area. **c** Nonbreeding season timing for each individual included in analyses across the study period. Each horizontal bar shows the timing (start and end) and duration of the nonbreeding season and each row is a unique individual. For individuals monitored for > 1 year, colored bars show the second year (circles) and third year (triangles) of monitoring

Ibis were captured and fitted with GPS transmitters at 20 sites in South Florida, USA (Fig. 1) [42]. Ibis in this region are common in urban settings, including public parks, lawns, golf courses, and residential areas. At urban locations, ibis can forage on human-provided food such as picnic scraps and bread thrown intentionally to feed ibis and other wildlife [41]. Ibis also inhabit natural habitats in South Florida, including restored wetlands, wetlands for wastewater management, and coastal areas (Fig. 1b). At night, they roost communally in trees at sites that differ from foraging locations [50]. Ibis in this population often disperse away from coastal areas in the breeding season, but some remain in coastal South Florida [42], and some have begun breeding at urban sites with large water bodies in the last 5 years (S. Hernandez, *pers. obs.*).

Ibis were captured in multiple seasons at sites designed to represent a gradient of urban land use [41]. At urban sites, individuals were captured with nylon slip-knot leg lassos and modified manually operated flip traps [51]. At natural sites, ibis were captured using mist nets with decoys. A subset of captured ibis were fitted with ﻿EcoTone Kite GPS-GSM trackers (http://www.ecotone-telemetry.com; North Star Science and Technology, Oakton, VA, USA) (Fig. 1a, b). Ibis were only fit with transmitters if the attachment was < 3% of the bird’s body mass. Tags collected GPS locations every 2 h during daylight hours and collected one location after sunset or before sunrise, because ibis usually roost in place at night [42].

### Tracking data

All analyses are based on GPS tracking data of 34 ibis between 15 October 2015 and 24 March 2018, using coordinates restricted to South Florida (Fig. 1a, b). For each of the GPS-tagged ibis, we subsetted data to include only the nonbreeding season, which is the time when most individuals in the population move within the study area. We defined the nonbreeding season based on a previous analysis of characteristic locations and movement patterns that define behavioral seasons [42], rather than from dates alone. The nonbreeding season usually began between September and November and ended in March, though there were some exceptions (Fig. 1c).

We separated data by bird-year for subsequent analyses, resulting in 61 tracks that included all the relocations of an individual bird in a single nonbreeding season. Many individuals (13) were tracked in only one nonbreeding season, 21 were tracked over two nonbreeding seasons and two were tracked over three (Fig. 1c). For each track, we normalized the fix rate to 2 h with a tolerance of 20 min and filtered out location errors by removing any points whose locations would produce an apparent speed of greater than 43 km/h (12 m/s, [52]) between subsequent relocations.

We used R Version 4.0.1 for all analyses [53] and the package *amt* for normalizing fix rates [54].

### Identifying habitat nodes

To distinguish unique sites that would be defined as habitat nodes in the spatial network, we identified clusters of GPS points. We calculated pairwise distances between all GPS fixes using an equidistant conic projection (parallels at 33°N and 45°N), then used a 650-m buffer to group points in close proximity. This 650-m buffer represents the expected foraging radius of ibis in this population [41]. After calculating the distance matrix, we assigned clusters starting with the point with the most neighbors (i.e., the point with the largest number of other points within 650 m). Any location within 650 m of this point was assigned to the first cluster. From the set of remaining points (i.e., those not in the first cluster), we then identified the point with the most neighbors, and assigned that point and its neighbors to the second cluster. We continued this process until only points with no neighbors remained; each of these was then assigned to its own cluster.

Some clusters were adjacent to one another, which could occur if foraging areas were large (e.g., part of a large wetland complex) or very close together (e.g., an urban park proximate to a golf course). Therefore, we joined clusters if their points were within 325 m of each other. We did so by buffering all points in each cluster by 325 m; if any points from other clusters fell within this buffer, the two clusters were joined. A node was defined as the polygon made up of all 325-m buffered points from that node. We selected this shorter distance for forming nodes because preliminary analysis indicated that larger clustering distances of 650 m would yield unrealistically large nodes (some > 15 km^2^). Nodes ranged in geographic area from 0.33 to 8.79 km^2^.

### Land cover and node characteristics

We extracted land cover data within each node. Land cover data was sourced from the 2016 Cooperative Land Cover (CLC Version 3.2) map for the state of Florida [55]. This dataset includes 279 land cover classes in the region, but we reclassified it to 12 classes that represent relevant differences in habitat for ibis: artificial impoundments/reservoirs, coastal, cropland, estuarine, forest, freshwater forested wetlands, freshwater non-forested wetlands, lakes, parks/zoos, scrub, urban, and urban open land. Using this reclassified map, we calculated the percent cover of each land cover class within each node.

We further simplified these 12 land cover classes using non-metric multidimensional scaling (NMDS) in three dimensions. The input variables in the NMDS analysis were the proportion of each node that consisted of each land cover class, where each node was a data point. In this analysis, we saw a gradient of human development along the first NMDS axis, with wetlands and scrub habitats having negative values and urban and other human-dominated habitats having positive values (Figure S1). Thus, in future analysis we used a node’s NMDS1 value as a proxy for urbanization.

We used R packages *raster*, *rgdal*, and *rgeos* for spatial analyses [56–58] and package *vegan* for NMDS analysis [59].

### Building networks

We built a network of habitat nodes using observed relocations of GPS-tracked individuals (Fig. 1a). We considered a pair of nodes to be connected if an individual ibis moved between those nodes in one time step (i.e., the 2-h fix interval with 20-min tolerance). This two-hour interval is roughly the gut-passage time of an ibis (fed pelletized food, [60]), and thus is relevant to the inter-site transport of microbes, nutrients and contaminants acquired during foraging. We built a weighted, directed network from these connections. Edge weights were calculated as the number of observed relocations between a pair of nodes across all bird-years. Node sizes were the total number of visits to each node. Note that this definition of node size differs from the total number of fixes at each node because consecutive fixes at the same node count as one visit; we selected this metric of node size because we were more interested in movements between nodes than in time spent at each node. For each node and edge, we also recorded the number of unique bird-years using that node or path. We clipped the network to include only nodes within the extent of − 80.52° to − 80° longitude and 26.25° to 27.5° latitude, to exclude 146 nodes. We also removed additional nodes (*n* = 107) with no observed connections to other nodes within the study area. This cropping also removed five bird-years from analysis (i.e., individuals who never entered the study area). The final network included 377 nodes over 56 bird-years. We used the *igraph* package for building networks and network analysis [61].

### Simulating null networks

**Fig. 2:**
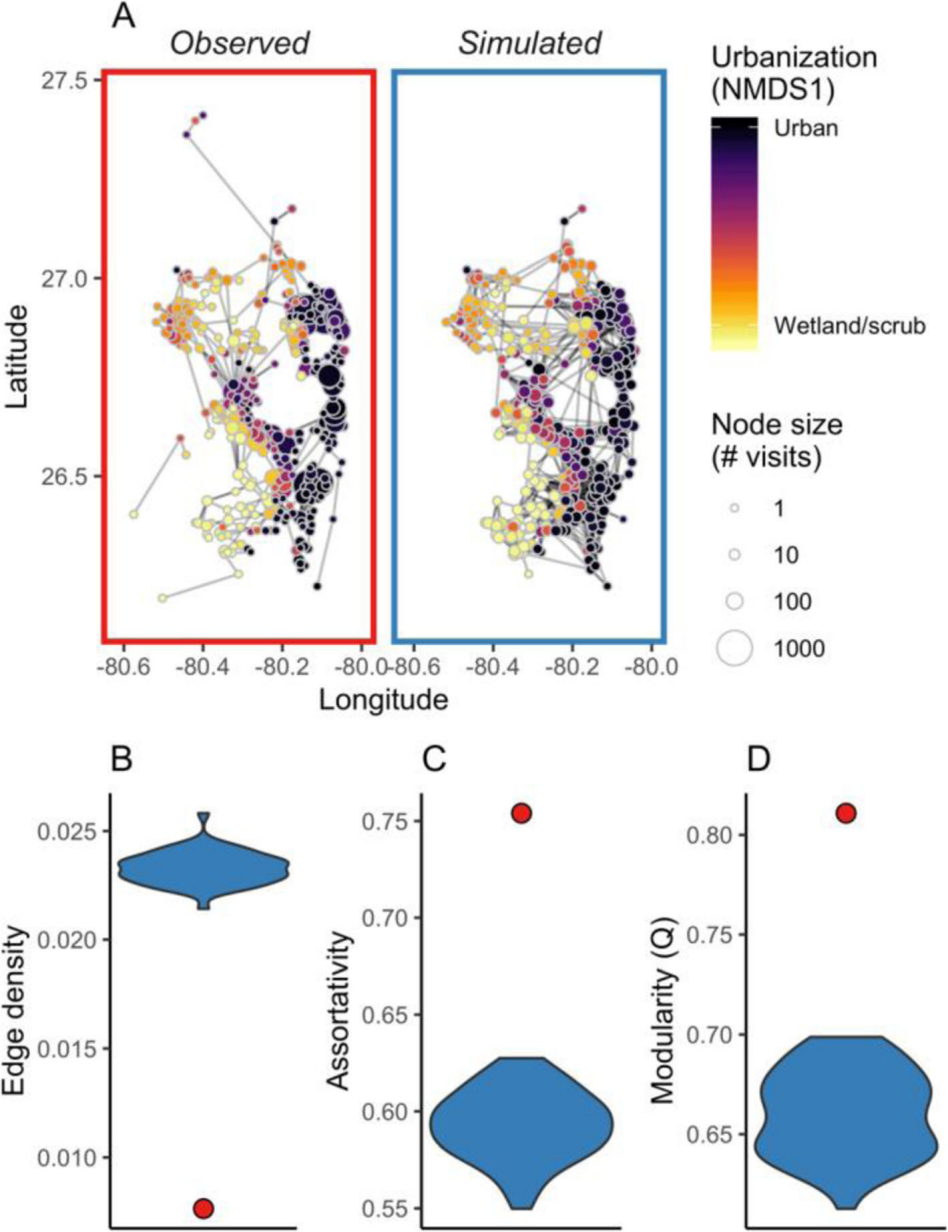
Networks and network properties. **a** Networks (visualized in geographic space) from observed tracking data and a representative simulated network. Points are colored by their urbanization score, measured as the first axis from the NMDS analysis of land cover proportions. Node size indicates total number of visits. **b**-**d** Properties of observed and simulated networks. Blue violin plots show the distribution of values for the random walk networks and red dots show the value for the observed network. **b** Edge density, the proportion of potential edges that are realized. Higher edge density indicates a more connected network. **c** Assortativity by site urbanization score (NMDS1). Higher assortativity indicates that nodes with similar properties are more connected with one another. **d** Modularity (Q) in the observed and simulated networks. Higher modularity indicates that the network can be divided more clearly into separate communities

To test how ibis movements determine network-level properties, and to account for spatial structure in our network, we created null models to compare with our observed data (Fig. 2a). To create these networks, we simulated random walks between nodes on the observed network (i.e., movements were not free on the landscape, but were constrained by node locations in the observed network). Each random walk was paired with an observed track (the full observed trajectory of an individual over a nonbreeding season); random walks began at the same starting point as the corresponding ibis track and were the same number of time steps as the observed track. In each time step, simulated ibis movements were based on two parameters derived from the tracking data: the probability of moving to a new node, and, if the individual moved, the probability of moving to each other node in the network. We used two different distributions for each of these parameters: one that pooled data across all individuals and one where parameters were based only on the specific track being simulated. These two distributions were designed to test the possibility that individuals are equal in their movement capacities and habitat preferences (pooled data) or that individuals differ in their movement patterns but still move independently of habitat type. In either case, the probability of moving to a new node was equal to the average proportion of 2-h intervals where (an) individual(s) moved among nodes. If an individual moved, the probability of moving between any pair of nodes was proportional to edge-length distribution (i.e., weighted interpatch distances) in the tracking data. Simulated networks thus accounted for the spatial arrangement of nodes on the landscape and ibis movement propensities, but not for site fidelity or habitat selection.

We repeated this process 50 times to create 50 sets of tracks that corresponded to the observed tracking dataset. We used these sets of simulated tracks to build 50 weighted, directed networks to compare to the observed network (Fig. 2a).

### Network properties and statistical analyses

We calculated network-level and node-level properties of both the observed and simulated networks (Table 1). At the node level, we analyzed degree centrality, betweenness centrality, and node size, each of which represented one aspect a node’s connectivity role (Table 1). Because our network was directed, we calculated degree centrality as out-degree, which is defined as the number of nodes that were visited by individuals departing from (not arriving at) a given node. We examined whether node land cover or spatial position predicted degree, betweenness, or node size using generalized linear models, where the predictor variables in each model were a node’s urbanization score (i.e., NMDS1), latitude, longitude, and quadratic terms for latitude and longitude (to model node geographic centrality). We centered and scaled all predictor variables to have a mean of 0 and a standard deviation of 1 to allow us to directly compare across parameter estimates. For degree and node size, we used a Poisson model with a log link because these measurements are counts; for betweenness, we used a Gaussian distribution and log-transformed the response variable.

At the network level, we calculated three metrics of network-scale connectivity (edge density, assortativity, and modularity; Table 1) for both observed and simulated networks. *Edge density* is the proportion of potential connections that are realized and represents network-scale connectivity [62]. We also calculated *assortativity*, the tendency of nodes with similar properties to be connected to one another [63]; we calculated assortativity for each land cover class separately, as well as for urbanization score (NMDS1, described above). We also identified modules, which are groups of nodes where a high proportion of edges exist within groups rather than between groups [63]. We tested three methods of module estimation: edge-betweenness [64], modularity optimization [65], and simulated annealing [46, 64], all calculated using the *igraph* package in R [61]. We compared the module membership of each node across the three methods; though each produced different numbers of modules, the module membership was qualitatively similar (Figure S2), so we used the method that produced the smallest number of modules (modularity optimization) in subsequent analyses. We calculated the modularity coefficient (*Q*) of the networks, which measures the strength of modularity and ranges from 0 to 1.

To test whether edge density, assortativity, or modularity differed between observed and simulated networks, we used linear models predicting density, assortativity, or modularity from network type (observed/simulated). For assortativity of each land cover class, we used a multivariate linear model predicting assortativity from network type, land cover class, and their interaction. To determine whether modules differed in their land cover, we used an analysis-of-variance (ANOVA) to identify whether module was a significant predictor of NMDS1.

### Variation in movement and habitat use across individuals

We used linear models to examine whether ibis differed in their use of nodes with different land covers. For this analysis, we compiled a dataset of all the nodes used by each individual and their NMDS1 scores. We used an ANOVA to identify whether individual was a significant predictor of NMDS1. For each individual, we also measured average urban habitat use by calculating a weighted average and weighted standard deviation of NMDS1 scores (i.e. NMDS1 score of each node, weighted by the number of times the individual was observed using that node) using the *Hmisc* package [66]. We examined whether urban habitat use was related to variation in habitat use (i.e., whether individuals using more urban habitats had more or less variation in their use of different land cover classes); to do so, we used a linear model with an individual’s weighted NMDS1 score as the response variable and the weighted standard deviation in NMDS1 score and its quadratic term as predictor variables.

To identify whether individuals differed in their importance for network connectivity, we removed each individual from the network one at a time. We calculated the differences in edge density and assortativity between the full and sparse network (i.e., a network with an individual removed). We used these differences to quantify the “connectivity role” of each individual in the network. We asked whether urban habitat use predicted an individual’s connectivity role using linear models, where the change in edge density or assortativity was the response variable and the individual’s weighted NMDS1 score and weighted standard deviation in NMDS1 score were predictor variables.

We repeated all of these analyses both at the level of the track (i.e., bird-year) and at the level of the individual to account for consistency and/or differences in individual node use across years.

## Results

### Network attributes

Ibis visited a network of 377 nodes across 56 bird-years within our study area (Fig. 2a). In our NMDS analysis, nodes separated along an urban-natural gradient; negative values in the first NMDS axis were associated with land cover classes that are less developed (i.e., freshwater non-forested wetlands, freshwater forested wetlands, and scrub) as well as croplands. Positive NMDS1 values were associated with land cover classes with higher anthropogenic influence, such as urban areas, parks/zoos, and urban open land (Figure S1). The stress value of the NMDS was 0.11, indicating a moderately good fit [67, 68]. We used the first NMDS axis as an urbanization score in subsequent analyses.

The edge density of the observed network was less than half of the mean edge density in the simulated networks (Fig. 2b). In other words, a significantly lower proportion of potential connections were realized than if birds were moving between nodes based on distance alone. Edge density was low in both observed and simulated networks (0.008 for observed and 0.020–0.023 for simulated networks), probably because we identified a large number of nodes but had movement data for only a small proportion of the local ibis population; thus, the relative values of edge density between the observed and simulated networks are more informative than raw values.

Assortativity was positive in both the observed and simulated networks and was higher in the observed than in the simulated networks, both for urbanization score and for each land cover class (Fig. 2c, Figure S3). Positive assortativity means that connections between nodes with similar urbanization scores or land cover classes were stronger than connections between nodes with different scores land cover classes, indicating clustering of nodes with similar land cover. This pattern was stronger in the observed than in the simulated networks, suggesting that spatial proximity alone did not explain assortativity. Assortativity was highest for urban and wetland land cover classes in both the observed and simulated networks (Figure S3).

We identified 19 modules in the observed network, with a modularity coefficient (*Q*) of 0.811. Within the nodes that made up each module, there was variation in urbanization score, but there was also distinct clustering along an urban/natural gradient (Figure S4). Module was significantly related to urbanization score (F = 23.09, *p* < 0.001), meaning that modules differed systematically in their level of urbanization. The observed network was significantly more modular than the simulated networks, measured using both *Q* (0.661 ± 0.021 SD in simulated networks) and the number of modules (range: 4–7 modules in simulated networks) (Fig. 2d, Figure S5).

These patterns, where connectivity was lower and assortativity was higher in the observed network, could arise either from individual specialization in habitat selection or from individual differences in movement propensities. However, even when we incorporated individual-specific movement parameters into null models, simulated networks were less assortative and more connected than the observed network, indicating that observed connectivity cannot be explained solely by variation in movement distances among individuals; rather, individuals differ in their use of habitat types.

### Node attributes

Each node was visited 33 times on average (SD: 109.7), but this distribution was right-skewed; the median number of visits was two, and 206 nodes (54%) were visited fewer than three times (Fig. 1d). In the simulated networks, the average number of visits per node was 14 (SD: 8.15) and this distribution was less skewed (median: 12.8). At the node level, degree was negatively associated with node urbanization score (Table S1). All measurements of centrality (i.e., degree, betweenness, and node size) tended to increase at the latitudinal center of the study area (i.e., negative association with latitude^2^). Neither node size nor betweenness was significantly related to node urbanization score. Geographic area was explained only by the number of visits to a node, and not by land cover or spatial position (Table S2), probably because node geographic area was determined by the method of identifying nodes from GPS data (i.e., nodes were created by aggregating and buffering ibis locations).

### Individual variation in movement patterns

**Fig. 3:**
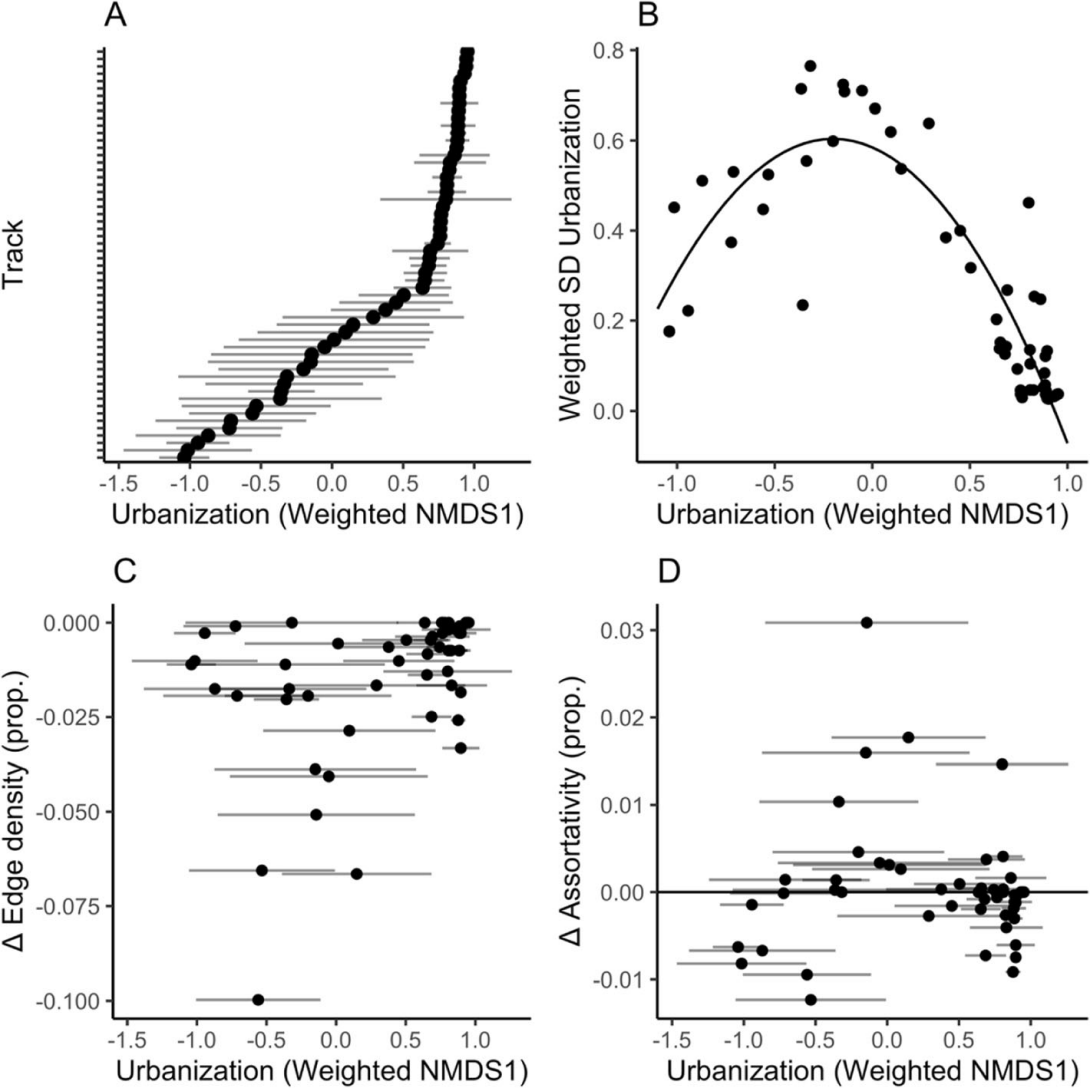
Individuals differ in their habitat use and their roles in network connectivity. **a** Weighted mean and weighted standard deviation of urbanization score (NMDS1) of all sites used by each individual bird-year. Individuals are sorted by their mean urbanization score. **b** Relationship between mean and standard deviation in weighted urbanization score. The curve shows the results from a linear model relating the two variables. **c** Relationship between an individual’s urbanization score and the change in edge density when they are removed from the network. Density can only decrease upon removal of an individual, so larger negative values indicate a larger influence of an individual on connectivity. **d** Relationship between an individual’s urbanization score and the change in assortativity when they are removed from the network. The horizontal line at y = 0 represents no change in assortativity

Ibis differed in the number and types of nodes they used (mean: 13 nodes per track, range: 1 to 77). Many birds were consistent in their use of specific land cover classes, particularly those that used primarily urban nodes (Fig. 3a). Individual (i.e., track or bird-year) was a significant predictor of node urbanization score (F = 18.2, *p* < 0.001). There was a hump-shaped relationship between the mean and variation in an individual’s habitat use, such that individuals with intermediate urbanization scores tended to have the highest variation in their urbanization score. Individuals using the most urban habitats had the lowest variation in their urbanization score (Fig. 3b).

When we removed individuals from the observed network, edge density decreased between 0 and 9.97% after the removal of a single track (Fig. 3c). This decrease was greatest at intermediate levels of urban habitat use, meaning that birds using primarily urban or wetland habitats had a smaller effect on edge density than did birds using both (i.e., negative effect of standard deviation in NMDS1, Table S3). Assortativity changed in both directions – either increasing or decreasing – upon removal of a single individual. Increases in assortativity tended to occur when individuals with intermediate urbanization scores and high variation in urbanization scores were removed (Fig. 3d, Table S3). These results were consistent, though slightly weaker, when analyzing individual birds (movement paths over multiple years) rather than tracks (path of an individual bird in a single year) (Figure S6).

## Discussion

Highly mobile animals can facilitate connectivity across space and between habitat types, but changes to movement in urban landscapes can alter connectivity. Our results suggest that connectivity in an urban-natural landscape mosaic depends on both the tendencies of individual birds and the characteristics of the sites they use. In particular, habitat specialization and site fidelity by individual ibis produced fewer connections and higher assortativity in the observed network. Ibis were less likely to make connections between nodes of different land cover classes than between nodes of the same class, and this pattern was stronger in our observed network than if ibis moved based on distance alone. Further, in the observed network, sets of nodes that were strongly interconnected (i.e., modules) tended to have similar land cover. The small number of birds that used both natural and urban sites contributed disproportionately to connectivity, and individuals that used the most urban sites were the most specialized in their habitat use, suggesting that continued urbanization could further reduce connectivity provided by this historically mobile species.

Individual specialization in habitat use, and in urban habitats in particular, contributed to low connectivity in our network analysis. Habitat specialization by individuals could arise if it provides fitness benefits [33, 69]. For example, urban environments can increase fitness in individuals with reduced antipredator responses [70] or favorable behavioral traits like boldness [13]. In this system and others, individuals captured in urban areas show isotopic signatures of anthropogenic food in their diet [41, 71], suggesting that urban specialization could increase food intake rates [72] (though it can have mixed effects on health outcomes: [41, 47]). In addition to these benefits, specialization could be maintained genetically if it reduces gene flow between urban and natural sub-populations; we focused on the nonbreeding season in this study, but individuals that use urban areas in the nonbreeding season can also be less likely to migrate long distances to breed (e.g., in wood storks: [73]) and ibis have recently begun nesting in urban areas in Palm Beach County (S. Hernandez, *pers. obs*). Our results show that, in addition to its potential benefits for individuals [33], habitat specialization has broader ecological effects by reducing overall connectivity.

The presence of habitat specialization in this population also calls into question the description of ibis as nomadic [38], at least in urban areas. The observed network differed from networks simulated by random walks for every metric we analyzed, which suggests that nomadic movements cannot be represented with random walks, and/or that urban ibis are not nomadic. For snail kites, a nomadic bird, distance alone does not explain movements [46], highlighting that random walks probably oversimplify nomadic movements. In addition, snail kite connectivity networks were not modular in the nonbreeding season, when they are nomadic in response to highly variable food resources [46]. In contrast, the observed ibis network was modular, suggesting that nomadism may not be an accurate description of ibis movements in this system at the scale of the nonbreeding season. The presence of stable resources in urban areas might reduce the benefits of nomadism [20, 72, 74]; comparing ibis movements in urban Palm Beach County with nearby populations in non-urban areas (e.g., in the Everglades) could clarify the role of urbanization in driving this non-nomadic movement.

Many individuals used primarily urban or natural sites, but a small number of habitat generalists connected urban and natural nodes. Ibis that used the most urban habitats had the smallest variance in their habitat use and were generally the least important for connectivity; the few individuals that visited the largest number of nodes had the greatest variation in their habitat use and played the largest role in connecting urban and natural nodes. These results indicate that individuals can be classified as natural specialists, urban specialists, or generalists (as in coyotes: [72]). Given their importance for maintaining connectivity, it is important to understand the abundance and characteristics of generalists in the population. Variation in movement and habitat use among individuals can stem from differences in phenotype, condition (e.g., body condition, reproductive state), and/or from environmental drivers [75–77]. If declines in natural site quality or the advantages of exploiting novel urban resources [77] were to make generalist individuals less common in the future, then connectivity could further decline. The outsize effect of generalists on connectivity is analogous to that of keystone individuals, which have a disproportionate impact on group dynamics [78], and whose removal affects reproductive success, social stability, and other population processes [78]. Understanding the ecology of generalist individuals can help predict whether their movement patterns will remain consistent as the environment changes, and therefore how other ecological processes will be affected.

The low connectivity that we identified between urban and natural areas could affect species interactions and community composition by limiting the dispersal of pathogens or propagules between sites of different types. For example, red squirrels are more likely to acquire paramyxovirus infections in conifer than in broadleaf forests [79], in which case low connectivity between conifer and broadleaf forests could reduce the risk of viral spread. At the same time, increases in site fidelity and decreases in movement can increase the size of epidemics in urban areas, particularly if urban sites accumulate high numbers of susceptible hosts [4]. Ibis sampled at urban sites have significantly higher prevalence of enteric *Salmonella* [43] and differences in their bacterial microbiome compared to ibis at natural sites [44], indicating that species interactions and pathogen transmission differ between urban and natural sites (see also [45]). The presence of generalist individuals in the population could maintain pathogen dispersal [80, 81], but the importance of highly mobile individuals for infection dynamics depends on both their abundance and the relative rates of movement and recovery [82], so the impact of generalists could be limited if they are much less common than habitat specialists.

Mobile animals are also important for nutrient transfer between habitats, so reduced connectivity in urban areas could affect ecosystem functioning. For example, in one study, approximately 86% of nutrients deposited by sharks at an atoll were derived from nearshore systems [83]; if these movements were to change in their frequency or location, this atoll could lose a substantial proportion of its nutrient inputs. In the ibis system, urban and natural diets differ in their nutrient composition [41] and phosphorous inputs from roosting birds can drive vegetation growth (e.g., tree islands in the Everglades [84]), so changes in diet and movement patterns in urban landscapes could have knock-on effects on landscape structure and ecosystem functioning.

The nonrandom nature of ibis movements we identified in this study highlights the importance of understanding drivers of movement for predicting connectivity. While it is well accepted that most movements are nonrandom, particularly at large spatial scales [85], connectivity studies often rely on random walks because other information is not available [86–88]. By incorporating movement data, we are able to more accurately illustrate how sites are connected by a focal species. We show that distance between sites is not the only component that needs to be considered for connectivity; if attempting to promote connectivity, managers should consider the characteristics that attract individuals to each site, and whether those characteristics vary across individuals. Our results showed no consistent relationship between node land cover and network centrality, suggesting that spatial position and/or other characteristics (e.g., disturbance, food availability) are more important in determining a site’s role in connecting the network. Efforts for maintaining connectivity are often based on establishing habitat corridors and removing barriers [88, 89], but for species whose movements are less limited by barriers it is necessary to develop other strategies that realistically incorporate the drivers and constraints on movement.

Understanding connectivity at the landscape scale is important for decisions about conservation and habitat management. To better inform these strategies, future studies could explore the characteristics of “stepping stone” patches that connect urban and natural areas, with the goal of managing these patches to increase or limit movements. Similarly, it is important to understand how landscape-scale connectivity and the strength of individual specialization impact gene flow, species interactions, and nutrient transfer. Last, future studies could examine how these patterns apply to larger spatial and temporal scales, such as whether habitat preferences are heritable and how they connect to longer-distance movements such as breeding dispersal.

## Conclusions

Urbanization and individual movement patterns can reduce connectivity, especially between urban and natural land cover classes. This reduction is true even for a highly mobile species whose movements are only minimally limited by habitat fragmentation. Instead, differences in habitat use between individuals reduce connectivity in a heterogeneous landscape, where individuals that use urban sites have the smallest role in connectivity because they rarely use non-urban sites. Lower connectivity could reduce the potential spread of pathogens or contaminants from urban to natural areas and the import of ecosystem services and beneficial nutrients or microbes from natural to urban areas [86].

## Supplementary Information

**Additional file 1: ** Supplementary Figures and Tables. Figures S1-S5 and Tables S1-S3.

## Data Availability

The datasets analyzed in this study and the code to generate results are available at Dryad (10.5061/dryad.bnzs7h492). Ibis tracking data are also available at Movebank (Kidd-Weaver et al. 2020, 10.5441/001/1.8ms50757).
