# Supplementary material for: Urban specialization reduces habitat connectivity by a highly mobile wading bird
Source: Mov Ecol. 2020 Dec 7;8:49. doi: 10.1186/s40462-020-00233-7 (PMC7720518; doi:10.1186/s40462-020-00233-7)
Supplement: Supplementary file 1 — Additional file 1: Supplementary Figures and Tables. Figures S1-S5 and Tables S1-S3. [file 40462_2020_233_MOESM1_ESM.docx]

## Supplementary figures and tables


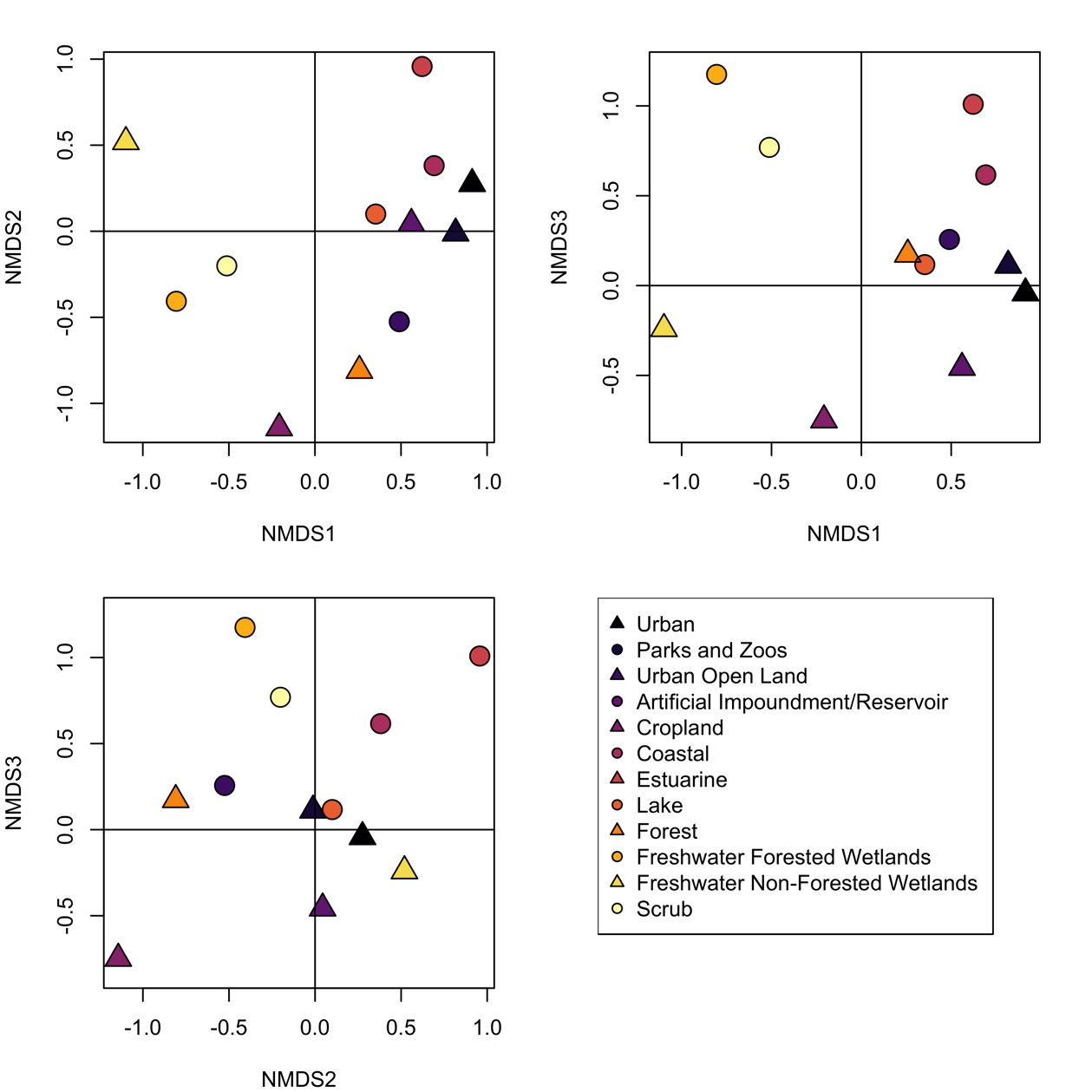


**Figure S1**: NMDS loadings for each of the 12 land cover categories. Colors are sorted by *a priori* categorizations of degree of human development; lighter colors show less developed habitats. Different shapes are included only to assist in identifying land cover classes but are not otherwise meaningful. Along the first NMDS axis, nodes separate out along a natural-urban gradient.


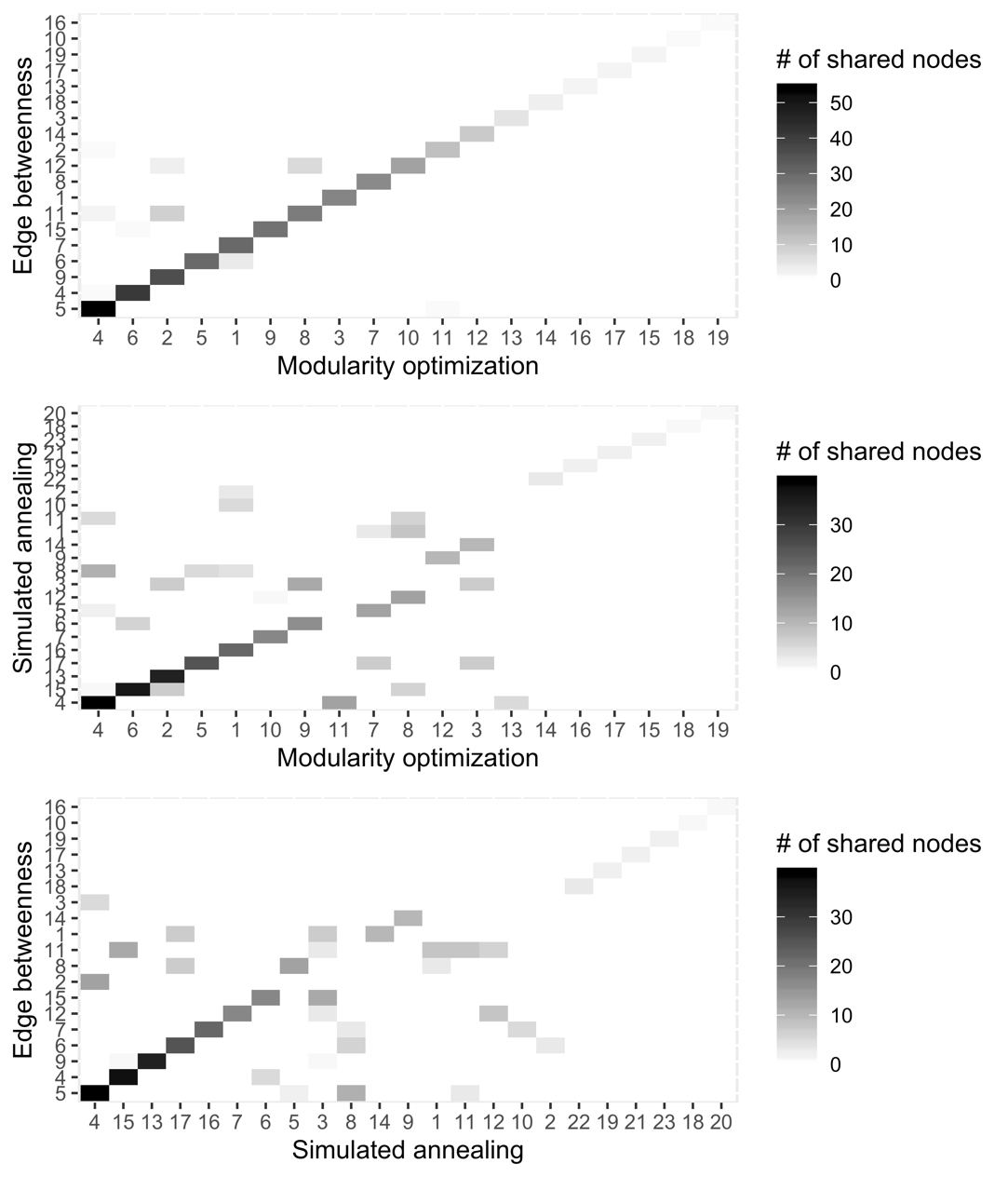


**Figure S2**: Module membership across methods. Each matrix shows the number of nodes shared between modules in each method. Modules are sorted by their number of shared nodes on each axis. (A) Modularity optimization vs. edge-betweenness. (C) Modularity optimization vs. simulated annealing. (B) Simulated annealing vs. edge-betweenness.


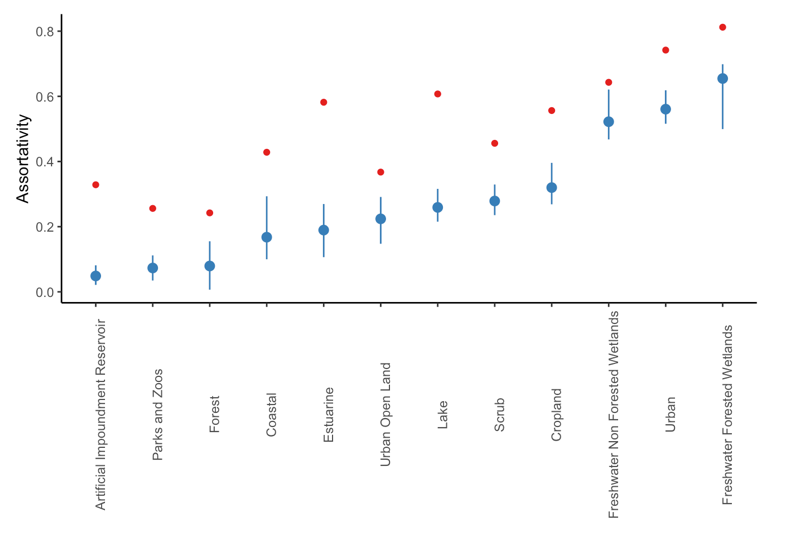


**Figure S3**: Assortativity for each land cover class. Red points show values for the observed network and blue points show the mean across 50 simulated networks. Blue lines show the range of values across the 50 simulated networks.


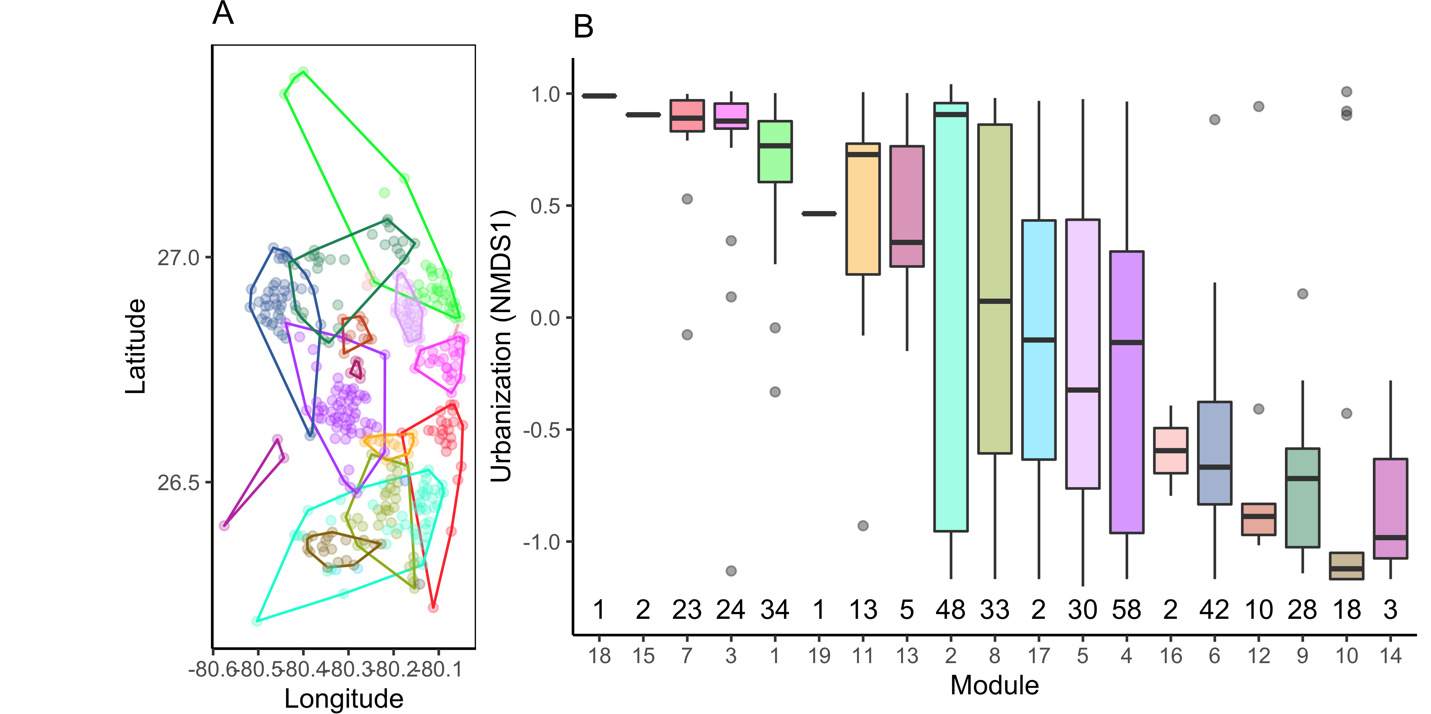


**Figure S4**: Module locations and characteristics. (A) Locations of all nodes in each module. Points show each node and polygons show convex hulls containing all nodes in a module. Modules with fewer than three points do not have a polygon drawn. (B) Urbanization score of the nodes in each module. Boxplots show the median and interquartile range of the data; whiskers extend out to 1.5* the interquartile range and outliers are shown as points. The numbers below each boxplot indicate sample size, i.e. the number of nodes in that module. The colors in the two panels correspond to the same module.


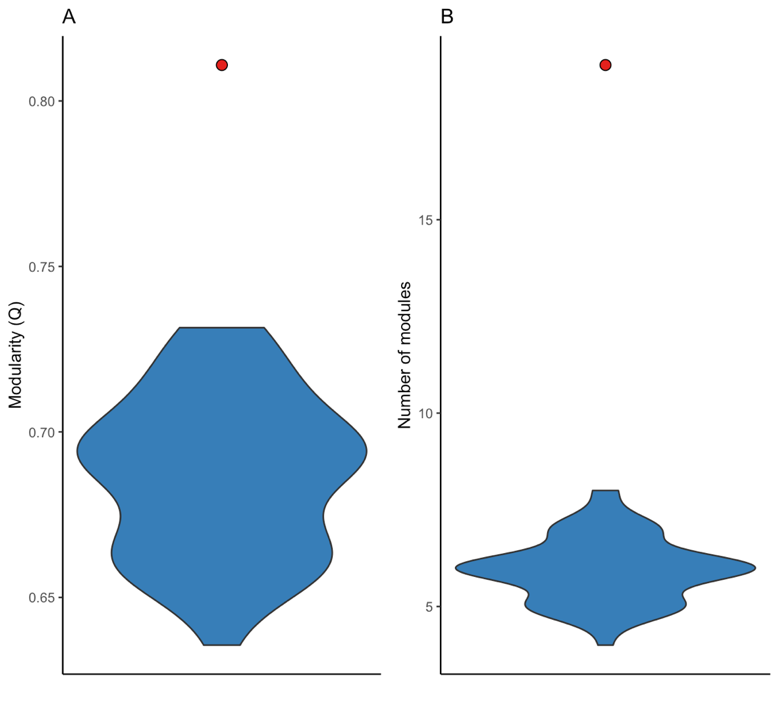


**Figure S5**: Modularity scores (A) and number of modules (B) in simulated and observed networks. Observed networks are the red points; violin plots show density distributions of values from 50 simulated networks.


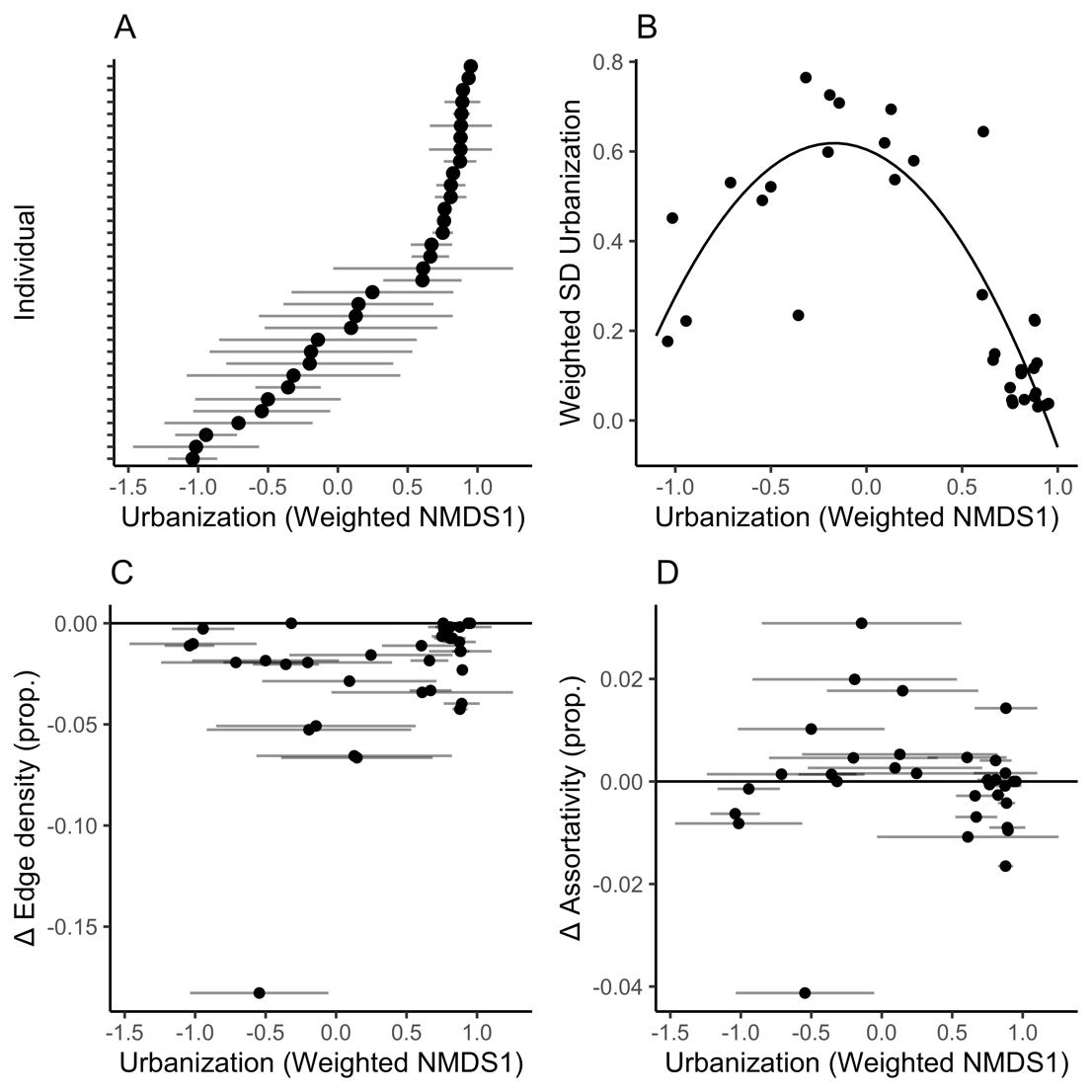


**Figure S6**: Individuals habitat use and connectivity role for individual birds rather than tracks (i.e. bird-years). (A) Weighted mean and weighted standard deviation of urbanization score (NMDS1) of all sites used by each individual. (B) Relationship between mean and standard deviation in weighted NMDS1 score. The curve shows the results from a linear model relating the two variables. (C) Relationship between an individual’s urbanization score and the change in edge density when they are removed from the network. Density can only decrease upon removal of an individual, so larger negative values indicate a larger influence of an individual on connectivity. (D) Relationship between an individual’s urbanization score and the change in assortativity when they are removed from the network. The horizontal line at y=0 represents no change in assortativity.

**Table S1**: Results from generalized linear models predicting metrics of node centrality and node size from spatial variables and urbanization score. All predictor variables were scaled to be centered at 0 with a standard deviation of 1, so larger parameter estimates indicate a stronger effect of a predictor variable. P-values below 0.05 are shown in bold.

**Degree centrality**

| **Variable** | **Estimate** | **SE** | **z** | **p** |
| --- | --- | --- | --- | --- |
| (Intercept) | 1.01 | 0.062 | 16.232 | **<0.001** |
| NMDS1 | -0.124 | 0.051 | -2.463 | **0.014** |
| lat | 0.074 | 0.042 | 1.768 | 0.077 |
| long | 0.212 | 0.051 | 4.146 | **<0.001** |
| lat^2^ | -0.261 | 0.044 | -5.911 | **<0.001** |
| long^2^ | 0.021 | 0.039 | 0.549 | 0.583 |

**Betweenness centrality**

| **Variable** | **Estimate** | **SE** | **z** | **p** |
| --- | --- | --- | --- | --- |
| (Intercept) | 5.313 | 0.306 | 17.389 | **<0.001** |
| NMDS1 | -0.266 | 0.251 | -1.062 | 0.289 |
| lat | 0.621 | 0.183 | 3.39 | **0.001** |
| long | 0.013 | 0.267 | 0.05 | 0.96 |
| lat^2^ | -0.406 | 0.153 | -2.65 | **0.008** |
| long^2^ | -0.271 | 0.195 | -1.391 | 0.165 |

**Node size**

| **Variable** | **Estimate** | **SE** | **z** | **p** |
| --- | --- | --- | --- | --- |
| (Intercept) | 1.536 | 0.153 | 10.073 | **<0.001** |
| NMDS1 | -0.055 | 0.125 | -0.438 | 0.661 |
| lat | 0.054 | 0.091 | 0.586 | 0.558 |
| long | 0.431 | 0.133 | 3.234 | **0.001** |
| lat^2^ | -0.24 | 0.076 | -3.139 | **0.002** |
| long^2^ | 0.137 | 0.097 | 1.409 | 0.16 |

**Table S2**: Results from a linear model predicting node geographic area (log-transformed area in km^2^) as a function of urbanization score (NMDS1), latitude, longitude, and number of visits to the node. P-values below 0.05 are shown in bold.

| **Variable** | **Estimate** | **SE** | **z** | **p** |
| --- | --- | --- | --- | --- |
| (Intercept) | 146.433 | 25583.505 | 0.006 | 0.995 |
| NMDS1 | 0.067 | 0.097 | 0.691 | 0.49 |
| lat | 94.325 | 50.477 | 1.869 | 0.062 |
| long | 34.939 | 636.684 | 0.055 | 0.956 |
| lat^2^ | -1.767 | 0.946 | -1.869 | 0.062 |
| long^2^ | 0.217 | 3.965 | 0.055 | 0.956 |
| # visits | 0.008 | 0.001 | 14.316 | **<0.001** |

**Table S3**: Results from generalized linear models predicting change in network properties following the removal of a single individuals. Both models used the proportional change in each network property (edge density or assortativity of NMDS1) as the response variable and the mean urbanization score (NMDS1) of all nodes used by an individual and the standard deviation of NMDS1 of all nodes used by an individual as predictor variables.

**Edge density**

| **Variable** | **Estimate** | **SE** | **z** | **p** |
| --- | --- | --- | --- | --- |
| (Intercept) | 0.002 | 0.002 | 0.956 | 0.344 |
| Mean NMDS1 | -0.001 | 0.001 | -0.837 | 0.406 |
| SD NMDS1 | 0.007 | 0.003 | 2.1 | 0.041 |

**Assortativity**

| **Variable** | **Estimate** | **SE** | **z** | **p** |
| --- | --- | --- | --- | --- |
| (Intercept) | -0.007 | 0.002 | -3.15 | 0.003 |
| Mean NMDS1 | 0.003 | 0.002 | 1.903 | 0.063 |
| SD NMDS1 | 0.015 | 0.004 | 3.882 | <0.001 |
